# An Invertebrate Hyperglycemic Model for the Identification of Anti-Diabetic Drugs

**DOI:** 10.1371/journal.pone.0018292

**Published:** 2011-03-30

**Authors:** Yasuhiko Matsumoto, Eriko Sumiya, Takuya Sugita, Kazuhisa Sekimizu

**Affiliations:** Laboratory of Microbiology, Graduate School of Pharmaceutical Sciences, The University of Tokyo, Tokyo, Japan; Universität Heidelberg, Germany

## Abstract

The number of individuals diagnosed with type 2 diabetes mellitus, which is caused by insulin resistance and/or abnormal insulin secretion, is increasing worldwide, creating a strong demand for the development of more effective anti-diabetic drugs. However, animal-based screening for anti-diabetic compounds requires sacrifice of a large number of diabetic animals, which presents issues in terms of animal welfare. Here, we established a method for evaluating the anti-diabetic effects of compounds using an invertebrate animal, the silkworm, *Bombyx mori*. Sugar levels in silkworm hemolymph increased immediately after feeding silkworms a high glucose-containing diet, resulting in impaired growth. Human insulin and 5-aminoimidazole-4-carboxamide-1-β-D-ribofuranoside (AICAR), an AMP-activated protein kinase (AMPK) activator, decreased the hemolymph sugar levels of the hyperglycemic silkworms and restored growth. Treatment of the isolated fat body with human insulin in an *in vitro* culture system increased total sugar in the fat body and stimulated Akt phosphorylation. These responses were inhibited by wortmannin, an inhibitor of phosphoinositide 3 kinase. Moreover, AICAR stimulated AMPK phosphorylation in the silkworm fat body. Administration of aminoguanidine, a Maillard reaction inhibitor, repressed the accumulation of Maillard reaction products (advanced glycation end-products; AGEs) in the hyperglycemic silkworms and restored growth, suggesting that the growth defect of hyperglycemic silkworms is caused by AGE accumulation in the hemolymph. Furthermore, we identified galactose as a hypoglycemic compound in jiou, an herbal medicine for diabetes, by monitoring its hypoglycemic activity in hyperglycemic silkworms. These results suggest that the hyperglycemic silkworm model is useful for identifying anti-diabetic drugs that show therapeutic effects in mammals.

## Introduction

The number of individuals diagnosed with type 2 diabetes mellitus, which is caused by insulin resistance and/or abnormal insulin secretion, is increasing worldwide [1], creating a strong demand for the development of more effective anti-diabetic drugs. Blood glucose levels are regulated by hormones such as insulin that regulate glucose uptake and metabolism in tissues throughout the body. Evaluation of the effects of anti-diabetic drugs thus requires the use of an animal model. The use of mammalian animals to screen for anti-diabetic drugs, however, is not only very expensive from an animal husbandry perspective, but also presents ethical problems in terms of animal welfare.

We previously reported that a silkworm infection model can be utilized to evaluate antibacterial and antiviral agents, and that there are a number of similarities in the pharmacokinetics of antibiotics between silkworms and mammals [2], [3], [4], [5], [6]. It is far less costly to rear silkworms than mammals, and a large number of larvae can be maintained in a small space. Screening of therapeutic agents can be easily performed with a large number of individual silkworms without the same ethical concerns involved in the use of mammals. Thus, we aimed to establish a method for evaluating the anti-diabetic effects of compounds using silkworms (Figure S1). Here we propose an invertebrate animal model of the disease utilizing the silkworm to evaluate the therapeutic effects of drugs.

## Results

### Immediate increase in sugar concentration in hemolymph of silkworms fed a high-glucose diet

**Figure 1:**
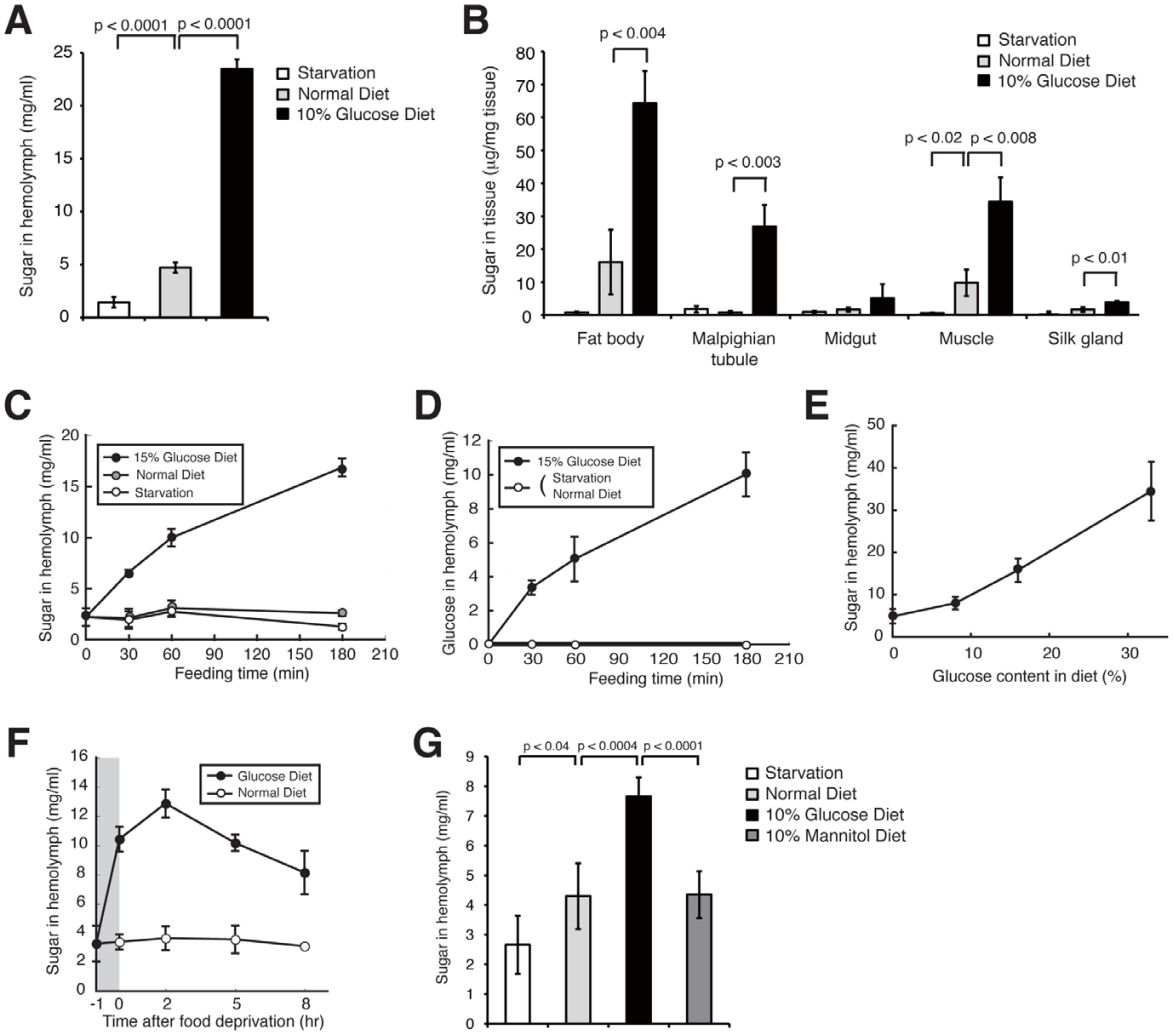
A high-glucose diet in silkworms increased hemolymph sugar levels. (A, B) Silkworms were fed a normal diet or 10% (w/w) glucose diet or fasted for 24 h, and then the hemolymph sugar level was determined (A). n = 5 per group. The sugar level in the fat body, malpighian tubule, midgut, muscle, and silk gland was determined (B). n = 3 per group. Data represents means ± SD. (C, D) Silkworms were fed a normal diet, 15% (w/w) glucose diet, or fasted for 0, 30, 60, or 180 min. Total sugar level (C) and glucose level (D) in the hemolymph were determined. n = 4–5 per group. (E) Hemolymph sugar levels in silkworms fed a normal diet, or an 8%, 16%, or 33% (w/w) glucose diet for 60 min. n = 3 per group. (F) Silkworms were fed a normal diet or 12% (w/w) glucose diet for 1 h (shown in gray) then fasted. Sugar level in hemolymph before feeding and at 0, 2, 5, or 8 h after fasting was determined. n = 5 per group. (G) Silkworms were fed a normal diet, 10% (w/w) glucose diet, 10% (w/w) mannitol diet, or fasted for 60 min, and hemolymph sugar levels were measured. n = 5 per group.

To establish a hyperglycemic silkworm model, we first evaluated the conditions required to induce hyperglycemia in silkworms. Silkworms fed a high-glucose diet (10% glucose-containing diet) for 1 day had a greater than 4-fold increase in the hemolymph sugar level compared with silkworms fed a normal diet (Figure 1A). The hemolymph sugar level of fasted silkworms was less than half that of silkworms fed a normal diet. The amount of sugar in the fat body, which corresponds to liver and adipose tissue in mammals, was also higher in silkworms fed a high-glucose diet than in silkworms fed a normal diet (Figure 1B). Increased sugar in the muscle and in the malpighian tubule, which corresponds to the mammalian kidney, was also observed in silkworms fed a high-glucose diet, although the amount of sugar was lower than that in the fat body (Figure 1B). The amount of sugar in the fat body of fasted silkworms was less than one-tenth that in silkworms fed a normal diet. Therefore, hemolymph and fat body sugar levels could be manipulated in silkworms by either feeding them a high-glucose diet or by fasting them.

**Figure 2:**
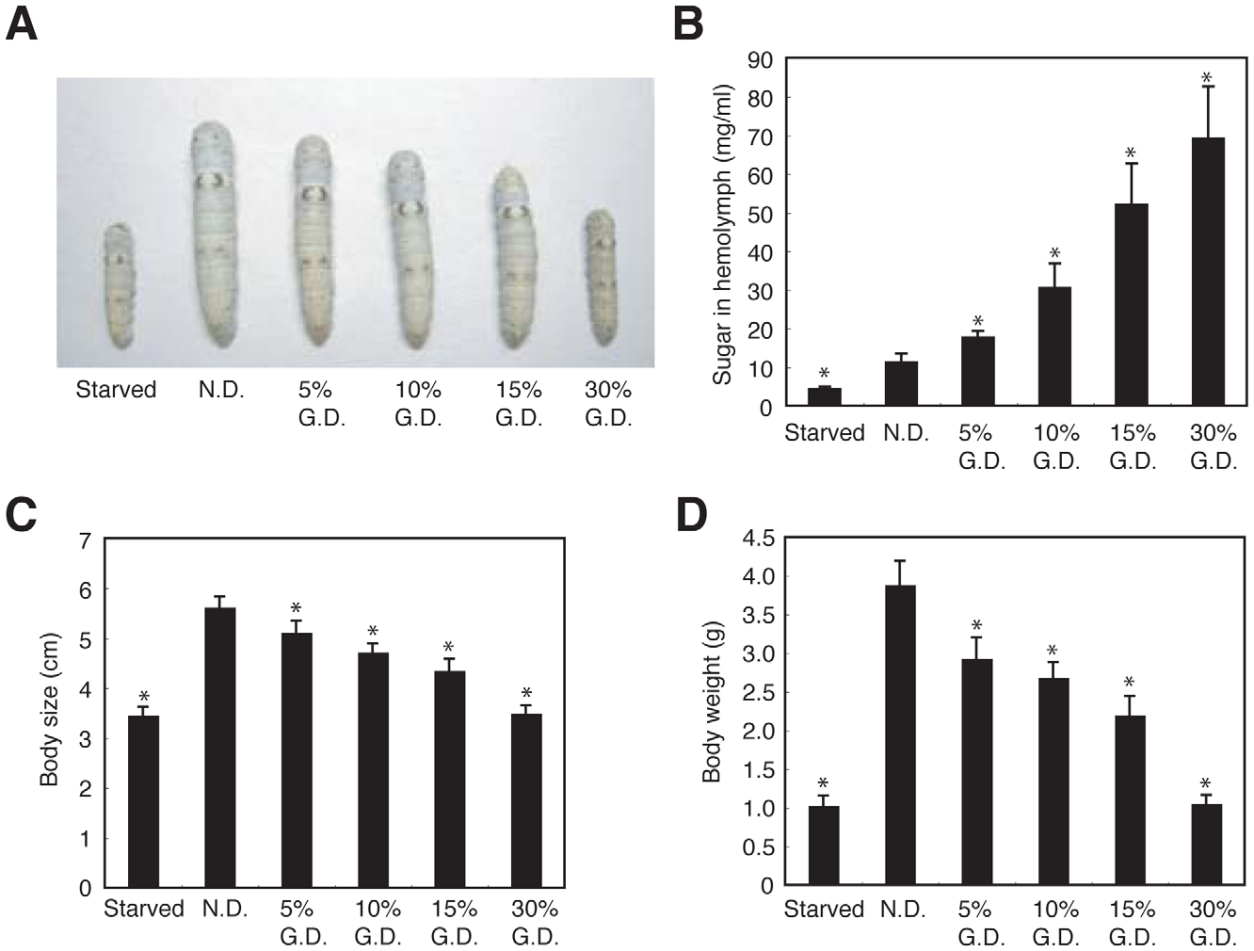
A high-glucose diet in silkworms inhibited growth. (A–D) Female silkworms were fed a normal diet (N.D.), a 5%, 10%, 15%, or 30% (w/w) glucose diet (G.D.), or fasted for 3 days. Sugar level in hemolymph (B), body size (A, C), and body weight (D) were determined. n = 8–15 per group. *p<0.0001 versus saline injected silkworms fed a normal diet (N.D.). Data are represented as means ± SD. In all panels, the statistical significance of the difference was evaluated using Student's *t* test.

**Figure 3:**
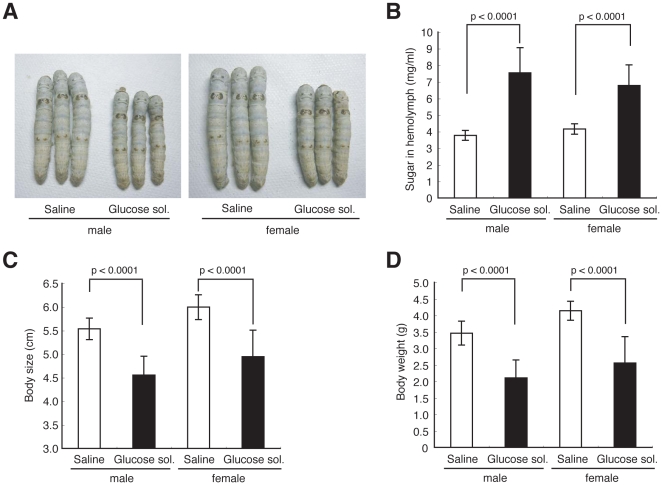
Growth inhibition of silkworms after injection of glucose. (A–D) Silkworms were fed a normal diet and injected with glucose solution (40%) or saline into the hemolymph every 12 h for 3 days. Hemolymph sugar level (B), body size (A, C), and body weight (D) were determined. n = 10–20 per group. Data represents means ± SD.

We then examined the time course of the increase in total sugar in the silkworm hemolymph during feeding with a high-glucose diet. Hemolymph sugar levels in silkworms fed a high-glucose diet increased 2-fold by 30 min, 4-fold by 60 min, and 6-fold by 180 min after feeding, respectively (Figure 1C). Hemolymph sugar levels in silkworms either fasted or fed a normal diet did not increase for up to 180 min. Glucose levels in the hemolymph were also measured using the glucose oxidase method. Glucose levels in the hemolymph of silkworms fed a high-glucose diet increased rapidly, whereas no glucose was detected in the hemolymph of silkworms either fed a normal diet or fasted (Figure 1D). We next tested whether hemolymph sugar levels increased according to the glucose content in the diet. Hemolymph sugar levels increased following intake of up to a 33% glucose diet without saturation (Figure 1E). These findings indicated that silkworms can be made hyperglycemic by feeding them a high glucose-containing diet. Hemolymph sugar levels in silkworms fed a high-glucose diet for 1 h began to decrease after fasting for the subsequent 2 h (Figure 1F). Hemolymph sugar levels were increased in silkworms fed a normal diet for 24 h. Fasting for the subsequent 12 h induced a drop in the hemolymph sugar level to that of continuously fasted silkworms (Figure S2). Silkworms fed a 10% mannitol diet did not show the increase in hemolymph sugar levels observed in silkworms fed a 10% glucose diet (Figure 1G). Thus, we assume that glucose is taken up in the silkworm midgut by a specific transporter-mediated system, thereby increasing the hemolymph sugar level. Together, these findings suggest that silkworms have a regulatory system for maintaining hemolymph sugar levels. Hemolymph sugar levels in silkworms fed a high glucose-containing diet increased more than 2-fold, indicating that we established a hyperglycemic model with silkworms. Diabetic patients generally suffer from several disorders due to hyperglycemia. We investigated whether hyperglycemia induced by feeding silkworms a high-glucose diet caused disorders. A high-glucose diet for 3 days increased hemolymph sugar levels (Figure 2B). Growth, in terms of body size and weight, was inhibited in both male and female hyperglycemic silkworms (Figure 2A, C, D, and Figure S3). Furthermore, administration of glucose into the silkworm hemolymph also increased sugar levels in the hemolymph and impaired growth (Figure 3).

### Human insulin and AICAR decrease sugar levels in silkworm hemolymph

**Figure 4:**
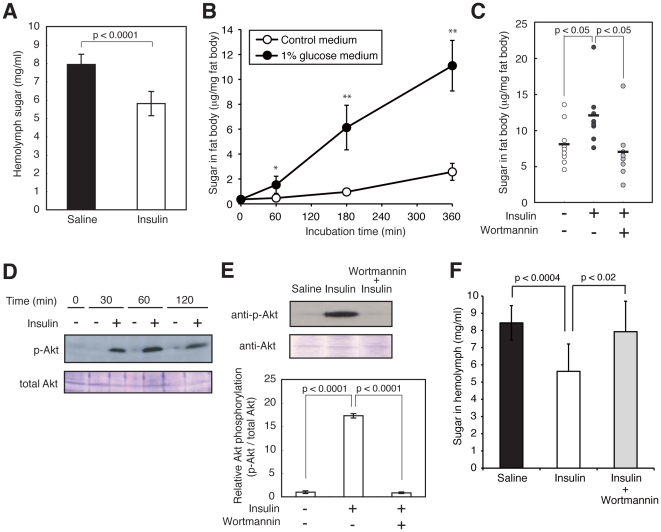
Decrease in the hemolymph sugar level of hyperglycemic silkworms and increase in Akt phosphorylation in the fat body by human insulin. (A) Silkworms were fed a 10% (w/w) glucose diet for 60 min. After removal of the diet, 50 µl of human insulin (3.5 mg/ml) was administered into the hemolymph of the hyperglycemic silkworms. Hemolymph sugar level was measured 6 h after injection. n = 6–7 per group. Data are shown as means ± SD. (B) Increase in total sugar in fat bodies cultured in insect medium containing 1% glucose. Isolated fat bodies from silkworm were cultured in Grace's insect medium containing 1% glucose for 0, 1, 3, or 6 h, and the amount of sugar in fat body was measured. n = 4–5 per group. *p<0.05, **p<0.001 versus control medium samples. (C) Isolated fat body from silkworm was cultured in Grace's insect medium containing 1% glucose with or without wortmannin (0.01 mM) for 30 min and further cultured with or without human insulin (final conc. 0.7 mg/ml) for 3 h. Total sugar in fat body was determined. n = 8–9 per group. (D) Isolated fat bodies from silkworm were cultured with human insulin (final conc. 0.6 mg/ml) in Grace's insect medium for 0, 30, 60, or 120 min. Fat bodies were homogenized and extracts were prepared. Total Akt and phosphorylated Akt were detected by immunoblot analysis. (E) Isolated fat body from silkworm was cultured in Grace's insect medium with or without wortmannin (0.01 mM) for 30 min and further cultured after adding human insulin (3 mg/ml) for 3 h. Immunoblots of total Akt and phosphorylated Akt (Top) and calculation of relative Akt phosphorylation (Bottom). n = 3 per group. Bottom data are shown as means ± SD. (F) Cancellation of the hypoglycemic effect of human insulin by co-administration of wortmannin. Silkworms were fed a 10% (w/w) glucose diet for 60 min. After removal of the diet, 50 µl of human insulin (3.5 mg/ml) with or without wortmannin (0.5 mM) was administered into the hemolymph of the hyperglycemic silkworms. Hemolymph sugar level was measured 6 h after injection. n = 9–10 per group. Data are shown as means ± SD. Statistical significance between groups was evaluated using Student's *t* test.

**Figure 5:**
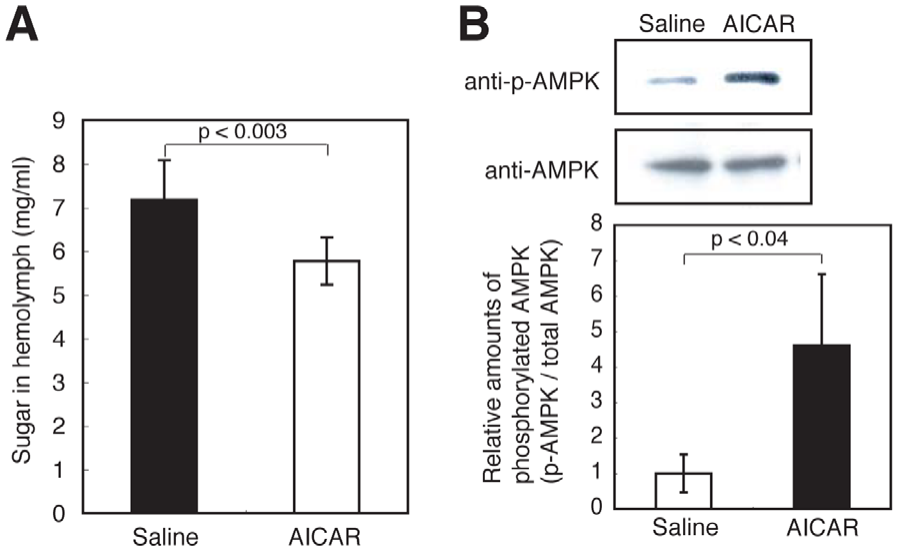
Decrease in the hemolymph sugar level of hyperglycemic silkworms and increase in AMPK phosphorylation in the fat body by AICAR. (A) Silkworms were fed a 10% (w/w) glucose diet for 60 min. After cessation of the diet, 50 µl of AICAR (4 mg/ml) was administered into the hemolymph of the hyperglycemic silkworms. Hemolymph sugar levels were measured 6 h after injection. n = 8–10 per group. Data are shown as means ± SD. (B) Western blot analysis of phosphorylated AMPK in fat body. Isolated fat body from silkworm was cultured with addition of AICAR (final conc. 0.8 mg/ml) for 2 h. Immunoblots of total AMPK and phosphorylated AMPK (Top) and calculations of relative AMPK phosphorylation (Bottom). n = 3 per group. Data at the bottom of the figure are shown as means ± SD.

**Figure 6:**
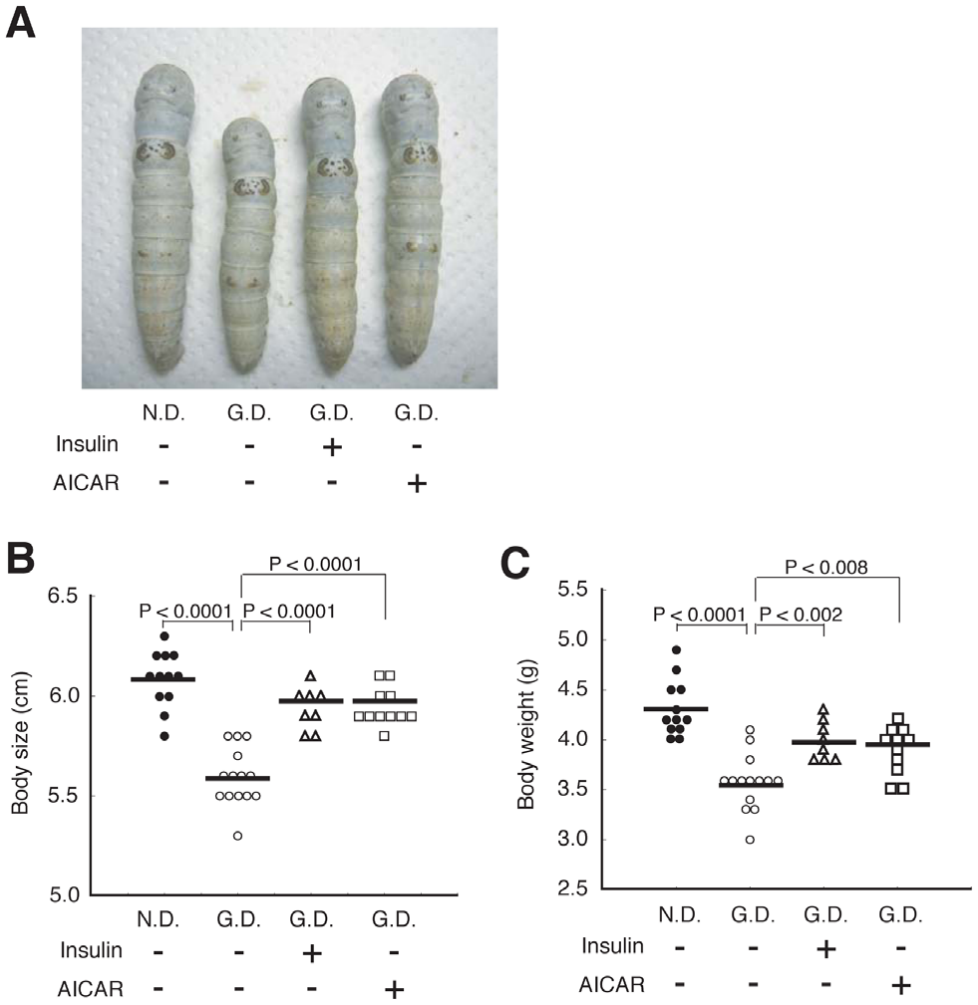
Repeated injections of human insulin and AICAR ameliorated growth defect in silkworm fed with high-glucose diet. (A–C) Silkworms were fed a normal diet (N.D.) or a 10% (w/w) glucose diet (G.D.) for 4 days. During the 4 days, silkworms were injected with 50 µl of human insulin (3.5 mg/ml), AICAR (4 mg/ml), or saline into the hemolymph every 12 h. Body size (A, B) and body weight (C) were measured. n = 8–14 per group. Bar represents mean. In all panels, the statistical significance of the difference was evaluated using Student's *t* test.

We next examined whether the hypoglycemic effect of anti-diabetic drugs can be evaluated using hyperglycemic silkworms. Insulin is a major therapeutic agent for patients with type I diabetes. The administration of recombinant human insulin decreased the hemolymph sugar level in silkworms fed a high-glucose diet (Figure 4A, and Figure S4A, B). In mammals, insulin enhances glucose uptake via Akt phosphorylation in tissues such as adipose tissue [7]. We tested whether human insulin enhanced glucose uptake into the fat body, the silkworm organ that corresponds to mammalian adipose tissue, in an *in vitro* culture system using isolated fat bodies. The amount of sugar in cultured fat bodies increased in a time-dependent manner after adding glucose to the medium (Figure 4B), indicating that isolated fat bodies have the capacity to take up glucose from the culture medium. Moreover, the addition of human insulin to the medium led to an increase in the total sugar and phosphorylated Akt in the fat bodies (Figure 4C–E). These effects by human insulin were inhibited by wortmannin, an inhibitor of phosphoinositide 3 (PI3) kinase (Figure 4C, E). Furthermore, the hypoglycemic effect of human insulin was blocked by the administration of wortmannin (Figure 4F). These results suggest that human insulin induces glucose uptake via the activation of phosphoinositide 3 kinase in the silkworm fat body, as in insulin-stimulated mammalian adipose tissue. Activation of the AMPK signaling pathway decreases blood glucose levels in mammals [8]. We examined whether AICAR, which activates AMPK, lowers sugar levels in silkworm hemolymph. AICAR injection decreased the hemolymph sugar level in hyperglycemic silkworms (Figure 5A). Moreover, the amount of phosphorylated AMPK was increased by the addition of AICAR to an *in vitro* culture system using isolated silkworm fat bodies (Figure 5B, and Figure S5). These results suggest that AICAR activates the AMPK pathway in the fat body and lowers sugar levels in silkworm hemolymph. We next examined whether human insulin or AICAR can restore the growth defect of hyperglycemic silkworms. In silkworms fed a 10% glucose diet for 4 days, both body size and weight were reduced compared to silkworms fed a normal diet. Under this condition, injection of human insulin or AICAR into the hemolymph of the hyperglycemic silkworms increased body size and weight compared to saline-injected controls (Figure 6). This finding suggests that human insulin and AICAR reverse the growth defect in hyperglycemic silkworms by lowering total sugar levels in the hemolymph. Therefore, the anti-diabetic effects of candidate drugs that activate the insulin signaling pathway and/or the AMPK signaling pathway can be evaluated using a silkworm hyperglycemic model.

### Increase in the amount of AGEs in the hemolymph of hyperglycemic silkworms

**Figure 7:**
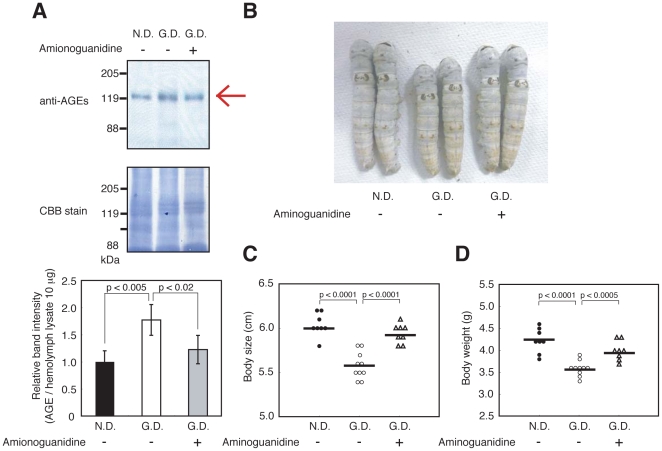
Increase in the amount of AGEs in the silkworm hemolymph after ingestion of a high glucose diet and decrease in AGEs after injection of aminoguanidine. (A) Western blot analysis of AGEs in the hemolymph. Silkworms were fed a normal diet (N.D.) or a 10% (w/w) glucose diet (G.D.) for 4 days. During the 4 days, silkworms were injected with 50 µl of aminoguanidine (10 mM) into the hemolymph every 12 h. The AGEs in hemolymph were determined by Western blot analysis with anti-AGE antibody. Hemolymph proteins were stained by Coomasie Brilliant Blue R-250. n = 3–4 per group. Top, immunoblots of AGEs. Middle, Coomasie Brilliant Blue staining. Bottom, calculations of relative band intensity. Bottom data are shown as means ± SD. (B–D) Body size (B, C) and body weight (D) were measured. n = 8–10 per group. Bar represents mean. In all panels, the statistical significance of the difference was evaluated using Student's *t* test.

The Maillard reaction is a series of nonenzymatic reactions, where carbonyl groups of reducing-sugars and amino groups of proteins form Schiff bases, which subsequently undergo Amadori rearrangements and oxidative modifications. The end result of these complex reactions is the formation of advanced glycation end-products (AGEs), which are considered to cause disorders in the tissues and blood vessels of diabetic patients. Recent studies suggest a correlation between the accumulation of AGEs and diabetic nephropathy [9], [10]. We examined whether AGEs are present in the hemolymph of hyperglycemic silkworms with impaired growth. A 120-kDa protein was detected in silkworm hemolymph using an anti-AGEs antibody by Western blot analysis (Figure 7A). The amount of the 120-kDa protein detected by anti-AGEs antibody was higher in silkworms fed a high-glucose diet (Figure 7A). Aminoguanidine, an inhibitor of the Maillard reaction, has therapeutic effects against cardiac hypertrophy and albuminuria in a diabetic rat model [11], [12]. Injection of aminoguanidine inhibited the increase of the 120-kDa AGEs in hyperglycemic silkworms (Figure 7A). We further tested whether aminoguanidine reverses/ameliorates the growth defect of hyperglycemic silkworms. Repeated injections of aminoguanidine in silkworms fed the high-glucose diet resulted in an increase of both body size and weight (Figure 7B–D). These results suggest that aminoguanidine ameliorates the growth defect of hyperglycemic silkworm by inhibiting AGE production in the hemolymph.

### Identification of galactose as an effective compound to decrease blood sugar levels

**Figure 8:**
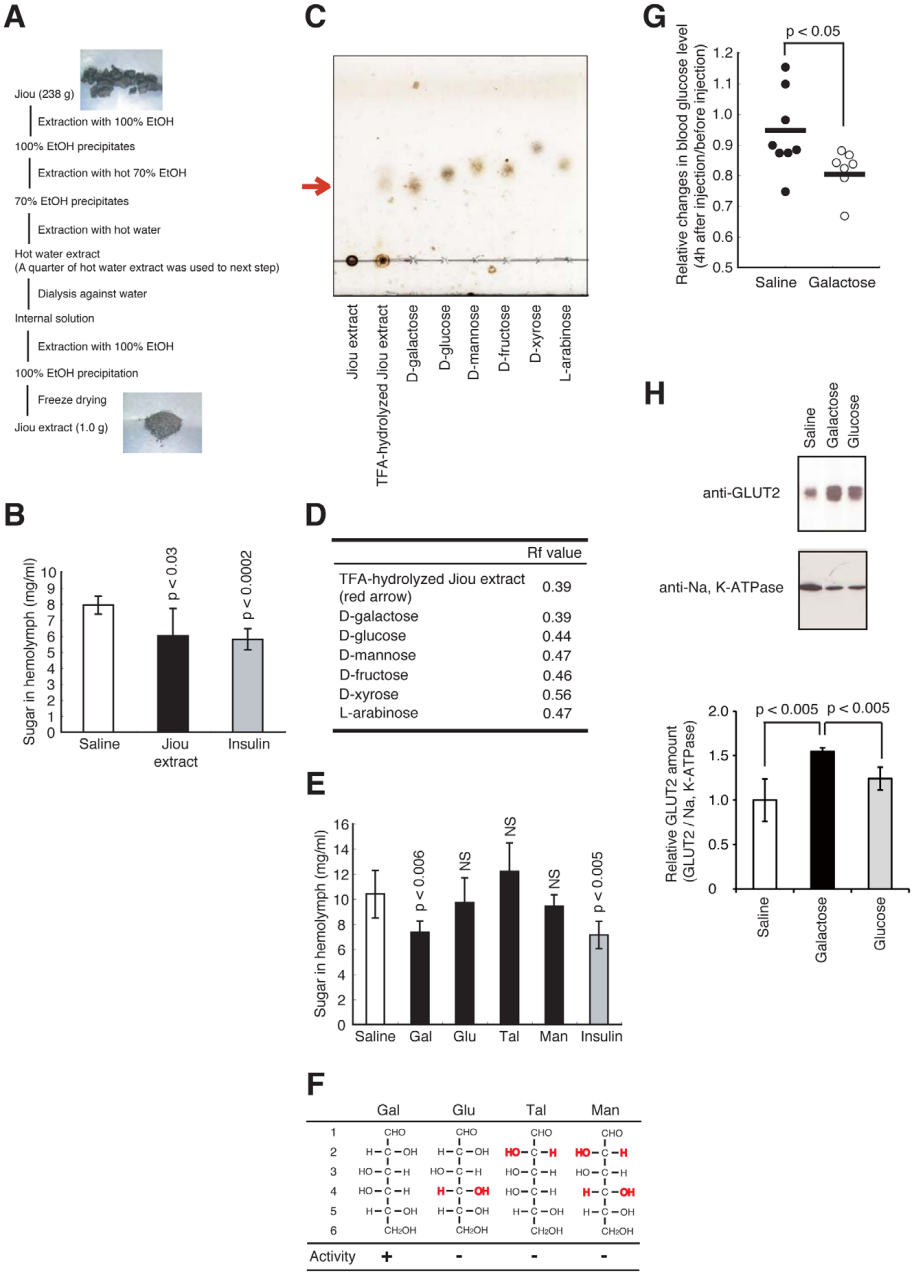
Identification of galactose as a hypoglycemic compound using the hyperglycemic silkworm model. (A) Preparation protocol for the jiou extract. (B) Silkworms were fed a 10% (w/w) glucose diet for 60 min. 50 µl of Jiou extract (1 mg/ml) or human insulin (3.5 mg/ml) was injected into the hemolymph of the hyperglycemic silkworms. The silkworms were fasted for 6 h and the sugar level in the hemolymph was determined. n = 6–7 per group. Data are shown as means ± SD. (C, D) The jiou extract was treated with TFA, and analyzed by TLC. Sugars were localized with 10% sulfuric acid solution. (E) Silkworms were fed a 10% (w/w) glucose diet for 60 min. 50 µl of D-Galactose (Gal), D-glucose (Glu), D-talose (Tal), D-mannose (Man), (1 mg/ml) or human insulin (3.5 mg/ml) was injected into the hemolymph of hyperglycemic silkworms. Silkworms were fasted for 6 h and sugar level in the hemolymph was determined. n = 6 per group. (F) Structure of sugar is shown by Fischer projection in the panel. Numbers shown on the left indicate the carbon positions of the sugar. Red hydroxyl group indicates the positions that differ from D-galactose. Activity represents the hypoglycemic effect. Data are shown as means ± SD. NS; not significant. (G) Galactose (10 mg/ml, 0.5 ml i.p.) was injected to streptozotocin induced-diabetic mice, and blood glucose level was determined after 4 h of fasting. Blood glucose levels in streptozotocin-induced hyperglycemic mice were measured (blood glucose 250–400 mg/dl) and then the mice were treated with either PBS or galactose solution. Four hours after administration and the removal of diet, the blood glucose levels were measured again. The data represent the blood glucose value after treatment divided by the blood glucose value before treatment of individual animals. In all panels, the statistical significance of the difference was evaluated using Student's *t* test. (H) Blood glucose levels in streptozotocin-induced hyperglycemic mice were measured (blood glucose 250–400 mg/dl) and then the mice were treated with either PBS, galactose (200 mg/kg mouse, i.p.), or glucose (200 mg/kg mouse, i.p.) solution. Two hours after administration and removal of the diet, the mice were killed and the membrane fraction in mouse liver was isolated. GLUT2 and Na, K-ATPase were detected by Western blot analysis with anti-GLUT2 antibody or anti-Na, K-ATPase antibody. Immunoblots of GLUT2 and Na, K-ATPase (Top) and calculations of relative GLUT2 (Bottom). n = 3–4 per group. Data at the bottom of the figure are shown as means ± SD. In all panels, the statistical significance of the difference was evaluated using Student's *t* test.

We next tested whether hyperglycemic silkworms are useful for identifying hypoglycemia-inducing compounds. Jiou, an herbal medicine, is considered to be effective for diabetic patients. Jiou has therapeutic effects in diabetic mouse and rat models [13]. A hot water extract of jiou reportedly has hypoglycemic activity in the streptozotocin induced-diabetic mouse model [13]. The active compound for the hypoglycemic effect in jiou, however, was not previously identified. We attempted to identify the hypoglycemia-inducing compound in jiou by using our silkworm diabetic model. Injection of the jiou extract decreased hemolymph sugar levels in hyperglycemic silkworms (Figure 8A, B). We presumed that the jiou extract contains a polysaccharide for two reasons; (i) the estimated sugar weight in the jiou extract was approximately half that of the dry weight, and (ii) the extract formed a precipitate after the addition of ethanol (Figure 8A). Therefore, we assumed that the polysaccharide in the jiou extract possessed a hypoglycemic effect. Thin layer chromatography (TLC) analysis of trifluoroacetic acid (TFA)-hydrolyzed materials from jiou revealed a single spot, which migrated to the same position as galactose. This spot was not observed when TFA-treatment was omitted (Figure 8C). Under this TLC condition, glucose, mannose, fructose, xylose, and arabinose migrated faster than galactose (Figure 8C, D). This finding suggests that the jiou extract contains galactose polymers. We next tested whether galactose shows hypoglycemic activity in hyperglycemic silkworms. Injection of galactose decreased the hemolymph sugar levels in hyperglycemic silkworms (Figure 8E). On the other hand, glucose, talose, and mannose, which are structural isomers of galactose, did not show this hypoglycemic effect (Figure 8E, F). We next tested whether galactose exerts hypoglycemic activity in a mammalian diabetic model. Intraperitoneal injection of galactose decreased the blood glucose levels in streptozotocin induced-diabetic mice (Figure 8G). Therefore, the hypoglycemic effect of galactose was demonstrated in hyperglycemic silkworms and in diabetic mice. To explore the molecular mechanism of the blood glucose reducing effect of galactose, we analyzed the expression level of glucose transporter 2 (GLUT2) in the liver of streptozotocin-induced diabetic mice. GLUT2 is expressed in the liver and facilitates glucose uptake [14]. GLUT2 levels in the membrane fraction prepared from the liver of diabetic mice were increased by galactose administration (Fig. 8H).

## Discussion

The findings of the present study demonstrated that hyperglycemia can be induced in silkworms by feeding a diet containing glucose. The total amount of sugar increased in the fat bodies of the hyperglycemic silkworms. An increase in hemolymph sugar in silkworms may lead to the uptake and accumulation of sugar in the fat bodies, similar to what is observed in the liver and adipose tissue in mammals. Trehalose, a dimer of two glucose molecules, is a major sugar in insect hemolymph and glucose is generally not detected in insect hemolymph. We detected glucose in the hemolymph of silkworms fed a high-glucose diet.

To examine whether glucose uptake is mediated by a specific transporter, we examined the dose response of glucose in the medium on sugar accumulation in the fat body. Excess glucose in the medium resulted in saturation of the sugar accumulation in the fat body (Figure S6). This finding suggests that at least in the fat body, sugar does not passively diffuse into the organ but is transported by a specific uptake system. Moreover, silkworms have a trehalose and glucose transporter, Tret1 (Trehalose transporter 1) [15]. In silkworms fed a normal diet, higher levels of Tret1 are expressed in muscle and in the fat body compared to the midgut, silk gland, or malpighian tubules [15]. The data we present here show that under normal diet conditions, sugar accumulation per unit weight of tissue is higher in muscle and in the fat body than in the midgut, silk gland, or malpighian tubules (Fig. 1B). The high sugar accumulation detected in organs expressing high levels of Tret1 indicates the possibility that sugar uptake is regulated by sugar transporters.

The administration of human insulin or AICAR decreased the hemolymph sugar level in hyperglycemic silkworms. This study is the first report demonstrating the possibility of evaluating the therapeutic effect of anti-diabetic drugs in an invertebrate hyperglycemic animal model. We also demonstrated that human insulin enhances the uptake of sugar into the fat body of silkworms by Akt phosphorylation via the activation of phosphoinositide 3 kinase. Therefore, the hypoglycemic effect of human insulin in hyperglycemic silkworms is due to activation of the insulin signaling pathway in silkworms, similar to mammals. Silkworms have bombyxin, a peptide hormone with structural similarity to human insulin [16]. Bombyxin increases phosphorylated Akt in silkworms [17]. Moreover, injection of glucose promotes the release of bombyxin into the hemolymph [18]. Silkworms might control the hemolymph sugar level by activating the insulin-signaling pathway with bombyxin. Activation of AMPK by AICAR also decreased hemolymph sugar levels in silkworms. Therefore, the anti-diabetic effects of candidate drugs that activate the insulin signaling pathway and/or the AMPK signaling pathway can be evaluated using a silkworm hyperglycemic model.

Impaired growth, a characteristic feature of hyperglycemic silkworms, may be due to the accumulation of AGEs. Injection of aminoguanidine, an inhibitor of the Maillard reaction, restored the impaired growth of hyperglycemic silkworms. There are established rodent models of diabetic complications, such as nephropathy, peripheral neuropathy, and retinopathy. Several months, however, are required to induce these complications. By comparison, the growth defect of hyperglycemic silkworms was observed within 3 days. Therefore, the hyperglycemic silkworm model may be highly useful for quickly evaluating the therapeutic effects of anti-diabetic drugs. Hemolymph sugar levels were not significantly increased in silkworms fed a diet with added olive oil or oleic acid (Figure S7A). Silkworms fed a high fat diet had low body weight and a low food intake (Figure S7B, C). These findings suggest that silkworms fed a high fat diet eat less, resulting in growth inhibition. Thus, compared to a high fat diet, a high glucose diet rapidly induces hyperglycemia in silkworms.

We previously reported similarities between silkworms and mammals with regard to drug toxicity and pharmacokinetics. 1) The therapeutic concentrations of antibiotics are similar in both a silkworm infection model and a mammalian infection model [2], [4]. 2) Intestinal uptake of several compounds is similar between silkworms and mammals [19]. 3) Like humans, silkworms have drug excretion mechanisms such as oxidization mediated by P450 and conjugation [3]. 4) The LD50 of toxic compounds is similar between silkworms and mammals [3], 5). Compounds with a relatively long half-life in mammals are also stable in silkworms [20]. Thus, we assume that silkworms could be useful for evaluating the drug toxicity and pharmacokinetics of compounds *in vivo*.

We screened for anti-diabetic agents using the hyperglycemic silkworm model. We found that an extract of jiou, an herbal medicine used to treat diabetes, has hypoglycemic effects when administered to hyperglycemic silkworms. Moreover, we demonstrated that galactose, a major component of the polysaccharides in jiou, had hypoglycemic activity in the silkworm diabetic model. Structural isomers of galactose, such as glucose, talose, and mannose did not have this hypoglycemic effect. In these galactose isomers, the position of the hydroxyl group(s) at C-4, C-2, or both differ from galactose. Therefore, the position of these hydroxyl groups in galactose is important for the hypoglycemic activity. Galactose also had a hypoglycemic effect in streptozotocin induced-diabetic mice. These findings suggest that the hyperglycemic silkworm model is useful for identifying anti-diabetic drugs that show therapeutic effects in mammals. To our knowledge, this is the first report that galactose has a hypoglycemic effect. Galactose is thought to have a hyperglycemic effect because it is isomerized to glucose in cells. The administration of excess amounts of galactose resulted in an increase in blood sugar levels in both mice and silkworms (data not shown). Accordingly, there is an optimal dose for galactose to exert its hypoglycemic activity. Some investigators reported that fructose and glucose have differential effects on food intake [21], [22]. On the other hand, the effects of galactose on the maintenance of blood sugar levels may differ from those of glucose. GLUT2 levels in the membrane fraction prepared from the liver of streptozotocin-induced diabetic mice were increased by galactose administration compared to that after injection of PBS or glucose. This result suggests that the specific action induced by galactose, upregulation of the GLUT2 level in the membrane and corresponding upregulation of glucose uptake into the liver, accounts, at least in part, for the blood glucose lowering effect. Understanding the molecular mechanism of the hypoglycemic activity of galactose may pave the way for the development of galactose derivatives as candidate anti-diabetic drugs.

The use of animals in experimental research should follow the guiding principles proposed by Russell and Burch in 1959, referred to as the “three R's” (Replacement, Reduction, and Refinement) [23]. Thus, screening for anti-diabetic drugs using a large number of mammalian animal models such as mice and rats is difficult because of ethical issues, especially in terms of animal welfare. Russell and Burch also introduced the concept of relative replacement, which recommends using invertebrate models instead of mammalian animals. Our newly-developed invertebrate hyperglycemic model using silkworms matches this concept.

## Materials and Methods

### Silkworm rearing conditions, glucose diet preparation, and injection methods

Fertilized eggs of silkworm, *Bombyx mori* (Hu·Yo x Tukuba·Ne; Ehime Sanshu), were kept in disposable plastic containers at 27°C. Hatched larvae were reared to the fifth instar on an artificial diet, SilkMate 2S, which contains antibiotics (Nosan Corporation), at 27°C. All experiments were performed using fifth-instar male larvae (0.9–1.0 g) fasted overnight during the fourth ecdysis, unless otherwise mentioned.

The glucose diet was prepared by mixing Silkmate 2S and D-glucose at the amounts indicated as the percentage of glucose in the total diet.

Injection experiments were performed as follows[24]. Sample solution (50 µl) was injected into the hemolymph at the second abdominal segment of the larva. Syringes (1 ml) and needles (27G×3/4) were purchased from Terumo.

### Sugar quantification

Hemolymph (20 µl) was collected from the larva through a cut on the first proleg ands mixed with 9 volumes of 0.6N perchloric acid. Precipitated proteins were removed by centrifugation at 3000 rpm for 10 min at 4°C. The supernatant (hemolymph extract) was diluted with the appropriate volume of distilled water for sugar quantification.

Total sugar in the hemolymph was determined using the phenol-sulfuric acid (PSA) method[25]. Hemolymph extract (100 µl) was mixed vigorously with 100 µl of 5% phenol aqueous solution, followed by vigorous mixing with 500 µl sulfuric acid, incubation at room temperature for 20 min, and absorbance at 490 nm was measured. Serially diluted glucose solution was used as a standard.

Glucose in the hemolymph was determined using the glucose oxidase method. Hemolymph extract (20 µl) was mixed with 400 µl of reaction solution (0.12 M sodium-phosphate buffer [pH 7.4] containing 4 U/ml glucose oxidase, 3 U/ml peroxidase, and 9 mM *o*-dianisidine), followed by vigorous mixing with 100 µl of 70% sulfuric acid solution, incubation at room temperature for 40 min, and absorbance at 530 nm was measured. Serially diluted glucose solution was used as a standard.

The fat body, isolated from the dorsolateral region of the larva, was rinsed in insect saline (130 mM NaCl, 5 mM KCl, and 1 mM CaCl_2_), and weighed. The fat body (1∼10 mg) was lysed in 50 µl of 30% KOH with heating at 90°C for 10 min. Distilled water (150 µl) and ethanol (300 µl; final 60%) were added and the mixture was incubated at 90°C for 10 min. The samples were incubated at 4°C overnight and centrifuged at 15,000 rpm for 3 min. The precipitate was dissolved in distilled water to give a concentration of 50∼100 mg fat body/ml by heating at 90°C for 10 min. The resulting fat body extract was used for sugar quantification by the PSA method. The amount of sugar in 1 mg of fat body was calculated.

### Chemicals

Recombinant human insulin was purchased from Wako and dissolved in 0.9% NaCl containing 0.1% acetic acid. Wortmannin was purchased from Calbiochem. AICAR was purchased from Toronto Research Chemicals Inc. Jiou was purchased from Uchida Wakanyaku. D-Glucose was purchased from Nacalai Tesque. D-galactose, D-mannose, and D-talose were purchased from Wako.

### *In vitro* fat body sugar uptake assay

The fat body (wet weight 2∼10 mg) was isolated from the dorsolateral region of the larva, rinsed in insect saline, and cultured in 200 µl Grace's insect medium supplemented with 1% glucose and antibiotics (penicillin and streptomycin) at 27°C for 30 min. Test sample solution (50 µl) was added to the culture medium, and the fat body was cultured and lysed and then the amount of sugar was determined using the PSA method.

### Immunoblot analysis

The fat body (wet weight 1∼10 mg) was isolated from the dorsolateral region of the larva, rinsed in insect saline, and cultured in 200 µl Grace's insect medium supplemented with 1% glucose and antibiotics (penicillin and streptomycin) at 27°C for 30 min with or without wortmannin. Test sample solution (50 µl) was added to culture medium and the fat body was cultured and then transferred to NP-40 lysis buffer (10 mM Tris/HCl [pH 7.5], 150 mM NaCl, 0.5 mM EDTA, 1 mM dithiothreitol, 1% NP-40, 10 mM NaF, and 1 mM Na_3_VO_4_) and lysed by sonication. The samples were centrifuged at 15,000 rpm for 3 min and proteins in supernatants were precipitated by trichloroacetic acid followed by centrifugation at 15,000 rpm for 15 min. The precipitates were washed twice with ice-cold ethanol, dissolved in a buffer with sodium dodecyl sulfate, heat-treated, and electrophoresed in a 12.5% polyacrylamide gel according to the method of Laemmli[26]. Proteins in the gel were electroblotted onto a polyvinylidene difluoride membrane (Millipore), probed with antibody, and detected using Western Lightning (Perkin-Elmer Life Sciences). The following antibodies were used for immunoblot analysis: rabbit polyclonal antibodies to total Akt, phosphorylated Akt, total AMPK, phosphorylated AMPK, Na, K-ATPase from Cell Signaling Technology, GLUT2 from ALPHA DIAGNOSTIC; and mouse polyclonal antibody to AGEs from Cosmo Bio Co., LTD.

For immunoblot analysis of hemolymph AGEs, silkworms were fed a normal diet or a 10% (w/w) glucose diet for 4 days. Aminoguanidine was injected into the hemolymph of the silkworms at 12-h intervals. The AGEs in hemolymph were detected by immunoblot analysis using anti-AGEs antibody and proteins were stained with Coomassie brilliant blue.

For immunoblot analysis of GLUT2 in mouse liver, the mouse liver membrane fraction was prepared as follows. Approximately 10 mg of mouse liver was harvested and cut into small pieces using scissors in Tris B (10 mM Tris/HCl (pH 7.4), 10 mM NaCl, 1.5 mM MgCl_2_) then centrifuged at 3300 rpm for 10 min. The supernatant was collected as liver extract. The extract was further centrifuged at 45,000 rpm for 1 h, and the resulting precipitate was dissolved by adding 50 µl of 1 M Tris base and used as the membrane fraction. GLUT2 and Na, K-ATPase in the membrane fraction of mouse liver were detected by immunoblot analysis using anti-GLUT2 and anti-Na, K-ATPase antibody.

Quantification of the amount of phosphorylated Akt or phosphorylated AMPK or GLUT2 was performed by densitometric scanning with Image Gauge software. The relative amount of phosphorylated Akt or phosphorylated AMPK or GLUT2 on total Akt or total AMPK or Na, K-ATPase was determined. The amount of AGEs was normalized to the lysate protein concentration.

### TLC analysis

Jiou extract (0.4 mg) was mixed with TFA solution (final 2 M) and incubated at 96°C for 2 h. The sample was dried by evaporation and dissolved in 50 µl water. The sample (5 µl) was spotted on a silica gel plate (Silica gel 60F254, Merck) and developed with a propanol solution (1-propanol∶water = 85∶15). The plate was sprayed with 10% sulfuric acid solution (sulfuric acid:ethanol = 10∶90) and heated to detect the spots.

### Streptozotocin-induced diabetic mouse

Mature male C57BL6/J mice (8 weeks of age) were purchased from SLC. Diabetes was induced by a single intraperitoneal injection of streptozotocin (150 mg/kg)[27]. Blood samples were collected from the tail vein 4–7 days after injection of streptozotocin and the blood glucose concentration was determined using a glucometer (Accu-Chek Aviva, Roche). Mice with a blood glucose level of 250 to 450 mg/dl were used to evaluate the hypoglycemic effects of test samples.

### Ethics Statement

All mouse protocols followed the Regulations for Animal Care and Use of the University of Tokyo and were approved by the Animal Use Committee at the Graduate School of Pharmaceutical Science at the University of Tokyo (approval number: P21-12).

### Statistical Analysis

Data are shown as means ± SD. Statistical significance between groups was evaluated using a two-tailed Student's *t* test. A p-value of less than 0.05 was considered statistically significant.

## Supporting Information

Figure S1 **Schematic illustration of the strategy for screening anti-diabetic agents using silkworms.**
(TIF)

Figure S2 **Increased hemolymph sugar levels in silkworms fed a normal diet followed by a decrease in hemolymph sugar levels induced by subsequent fasting.** Silkworms were fed a normal diet for 24 h (shown in gray), then fasted. The hemolymph sugar level of silkworms before feeding, 12 or 24 h after feeding, or fasted for 12 or 24 h was determined. n = 5 per group. Data represents means ± SD.
(TIF)

Figure S3 **Growth inhibition by feeding a high glucose diet in male silkworms.** (A–D) Male silkworms were fed a normal diet (N.D.), a 5%, 10%, 15%, 30% (w/w) glucose diet (G.D.), or fasted for 3 days. Body size (A, B), body weight (C), and sugar level in hemolymph (D) were determined. n = 7–10 per group. Data represents mean±SD. *p<0.0001 versus saline injected silkworms fed a normal diet (N.D.).
(TIF)

Figure S4 **Decrease in total sugar in hemolymph after injection of human insulin.** (A) Silkworms were fed a 10% (w/w) glucose diet (G.D.) for 60 min (indicated by gray background) then fasted. 50 µl of human insulin (2 mg/ml) was injected into the hemolymph of the hyperglycemic silkworms, and hemolymph sugar levels were measured 0, 1, 3, and 6 h after injection. n = 5–7 per group. Data represents mean ± standard deviation. *p<0.05 versus saline injected silkworms fed a glucose diet (G.D.). (B) Silkworms were fed a 10% (w/w) glucose diet for 60 min. After cessation of the diet, serially diluted human insulin (0.005–0.5 mg/g larva) was injected into the hemolymph of the hyperglycemic silkworms. Hemolymph sugar levels were measured 6 h after injection. n = 8–10 per group.
(TIF)

Figure S5 **Stimulation of AMPK phosphorylation in the fat body by AICAR.** Isolated fat bodies from silkworm were cultured with AICAR (final conc. 0.8 mg/ml) in Grace's insect medium for 0, 60, or 120 min. Fat bodies were homogenized and extracts were prepared. Total AMPK and phosphorylated AMPK were detected by immunoblot analysis.
(TIF)

Figure S6 **Effect of glucose concentration in the culture medium on total sugar in the fat body.** Isolated fat body from silkworms was cultured in Grace's insect medium containing 0%, 0.5%, 1.0%, or 2.5% glucose for 3 h, and the amount of sugar in the fat body was measured.
(TIF)

Figure S7 **Effect of a high fat diet in silkworms.** (A–C) Silkworms were fed a normal diet (N.D.); a 7.5%, 15%, or 30% (w/w) olive oil-containing diet; or a 7.5% or 15% (w/w) oleic acid containing diet for 1 day. Sugar levels in the hemolymph (A), body weight (B), and food intake (C) were determined. n = 5 per group. Data represents mean±SD. The statistical significance of the difference was evaluated using Student's *t* test. p: P value versus silkworms fed a normal diet (N.D.).
(TIF)
