# Supplementary figures and images for: An Invertebrate Hyperglycemic Model for the Identification of Anti-Diabetic Drugs
Source: PLoS One. 2011 Mar 30;6(3):e18292. doi: 10.1371/journal.pone.0018292 (PMC3068166; doi:10.1371/journal.pone.0018292)

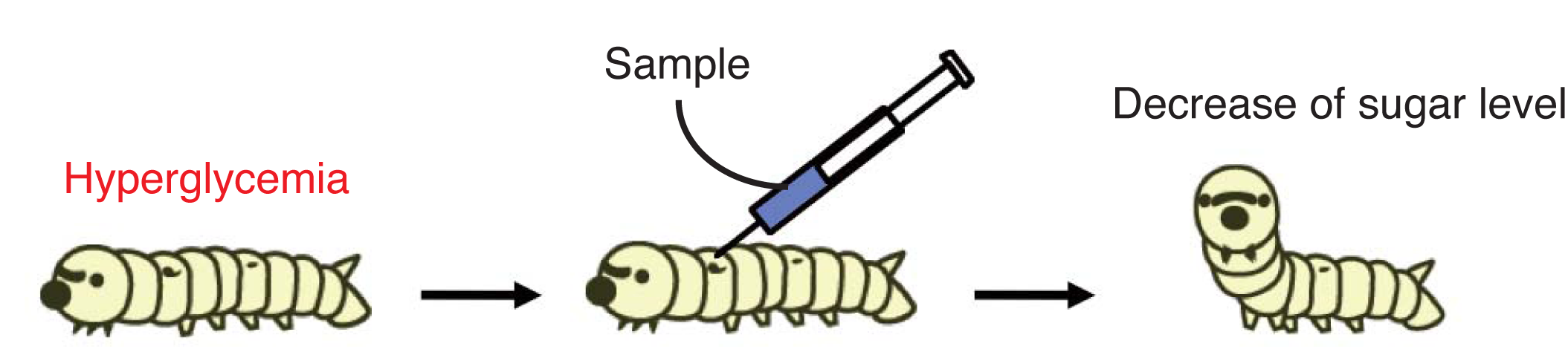

Supplement: Figure S1 — Schematic illustration of the strategy for screening anti-diabetic agents using silkworms. (TIF) [file pone.0018292.s001.tif]

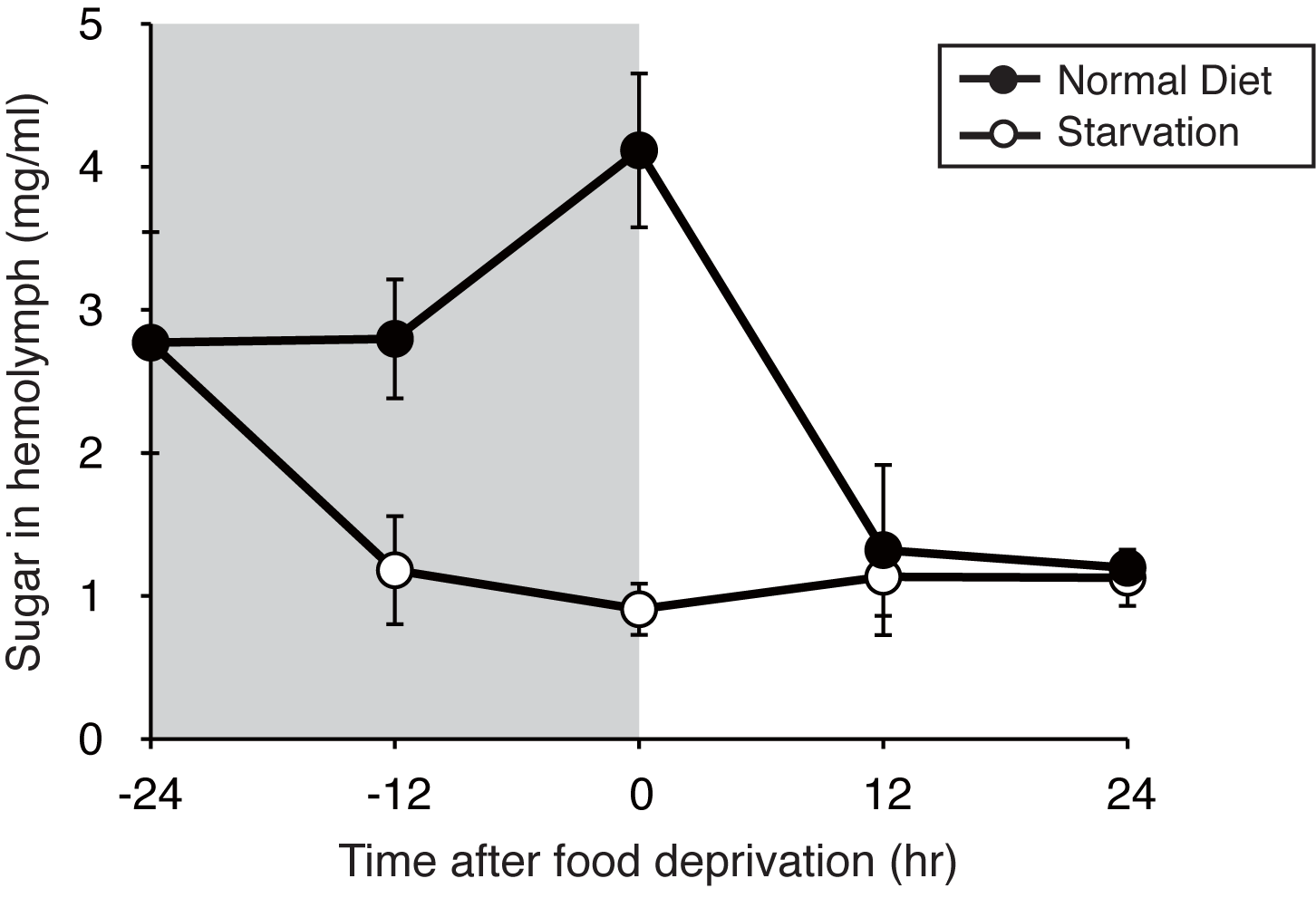

Supplement: Figure S2 — Increased hemolymph sugar levels in silkworms fed a normal diet followed by a decrease in hemolymph sugar levels induced by subsequent fasting. Silkworms were fed a normal diet for 24 h (shown in gray), then fasted. The hemolymph sugar level of silkworms before feeding, 12 or 24 h after feeding, or fasted for 12 or 24 h was determined. n = 5 per group. Data represents means ± SD. (TIF) [file pone.0018292.s002.tif]

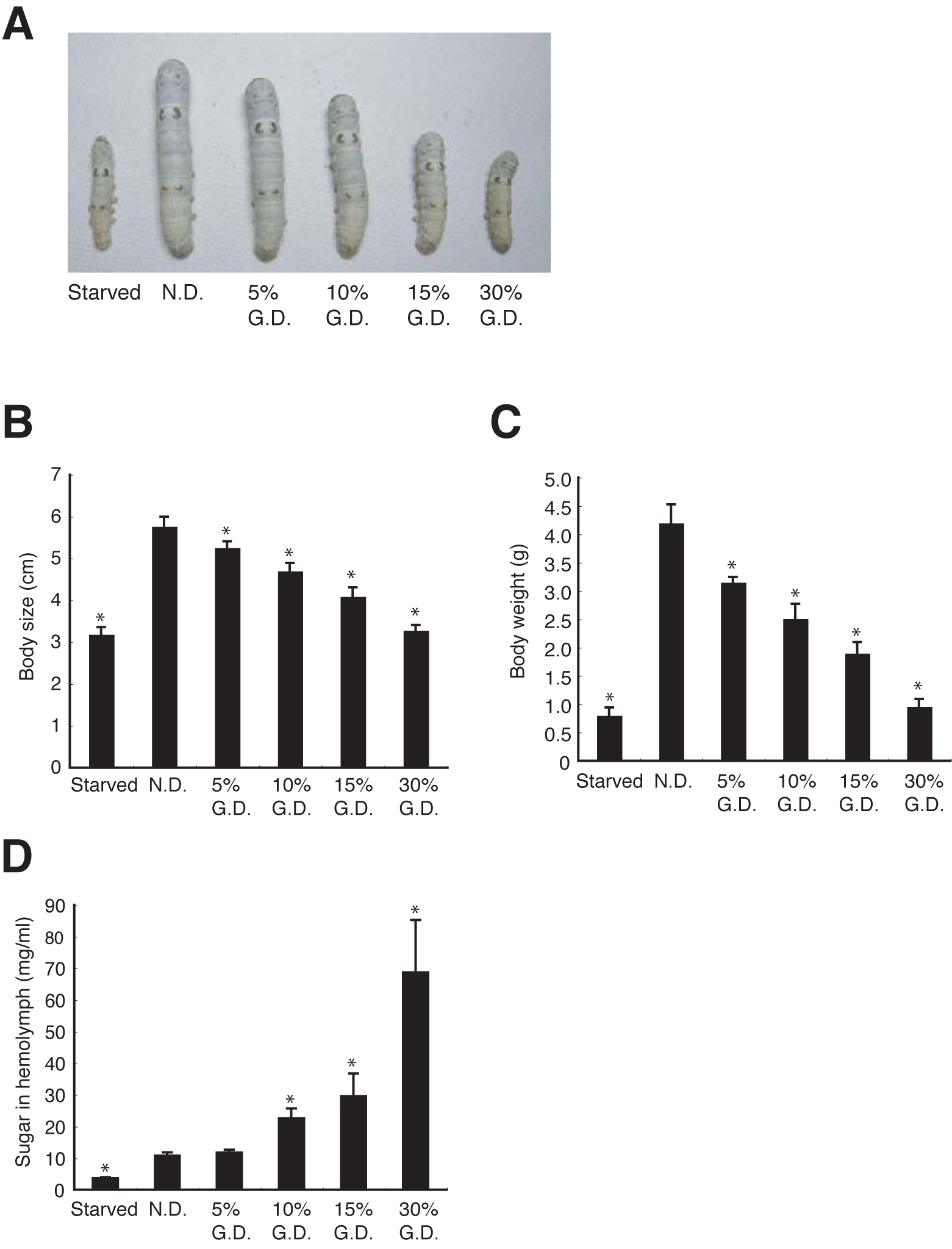

Supplement: Figure S3 — Growth inhibition by feeding a high glucose diet in male silkworms. (A–D) Male silkworms were fed a normal diet (N.D.), a 5%, 10%, 15%, 30% (w/w) glucose diet (G.D.), or fasted for 3 days. Body size (A, B), body weight (C), and sugar level in hemolymph (D) were determined. n = 7–10 per group. Data represents mean±SD. *p<0.0001 versus saline injected silkworms fed a normal diet (N.D.). (TIF) [file pone.0018292.s003.tif]

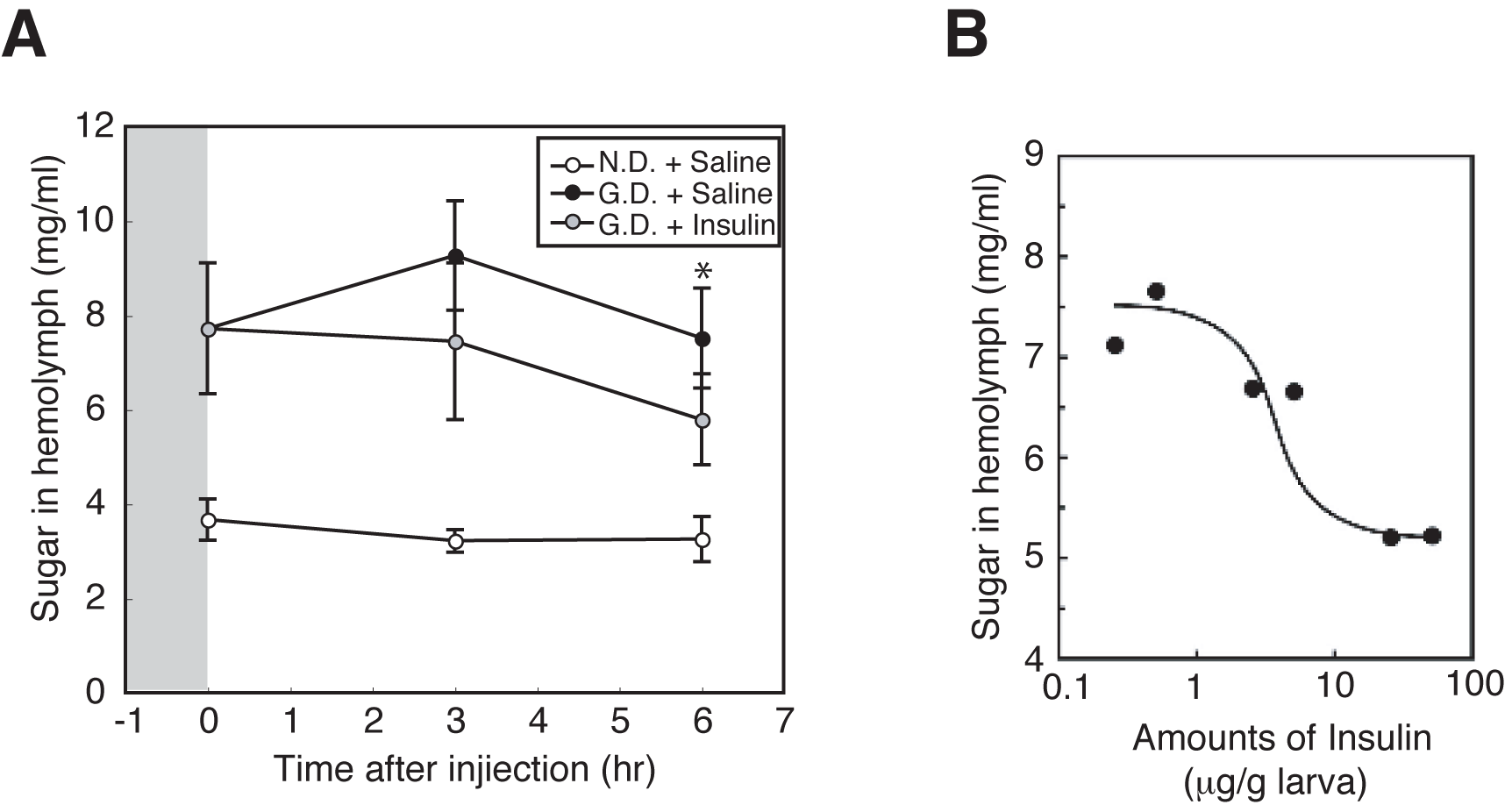

Supplement: Figure S4 — Decrease in total sugar in hemolymph after injection of human insulin. (A) Silkworms were fed a 10% (w/w) glucose diet (G.D.) for 60 min (indicated by gray background) then fasted. 50 µl of human insulin (2 mg/ml) was injected into the hemolymph of the hyperglycemic silkworms, and hemolymph sugar levels were measured 0, 1, 3, and 6 h after injection. n = 5–7 per group. Data represents mean ± standard deviation. *p<0.05 versus saline injected silkworms fed a glucose diet (G.D.). (B) Silkworms were fed a 10% (w/w) glucose diet for 60 min. After cessation of the diet, serially diluted human insulin (0.005–0.5 mg/g larva) was injected into the hemolymph of the hyperglycemic silkworms. Hemolymph sugar levels were measured 6 h after injection. n = 8–10 per group. (TIF) [file pone.0018292.s004.tif]

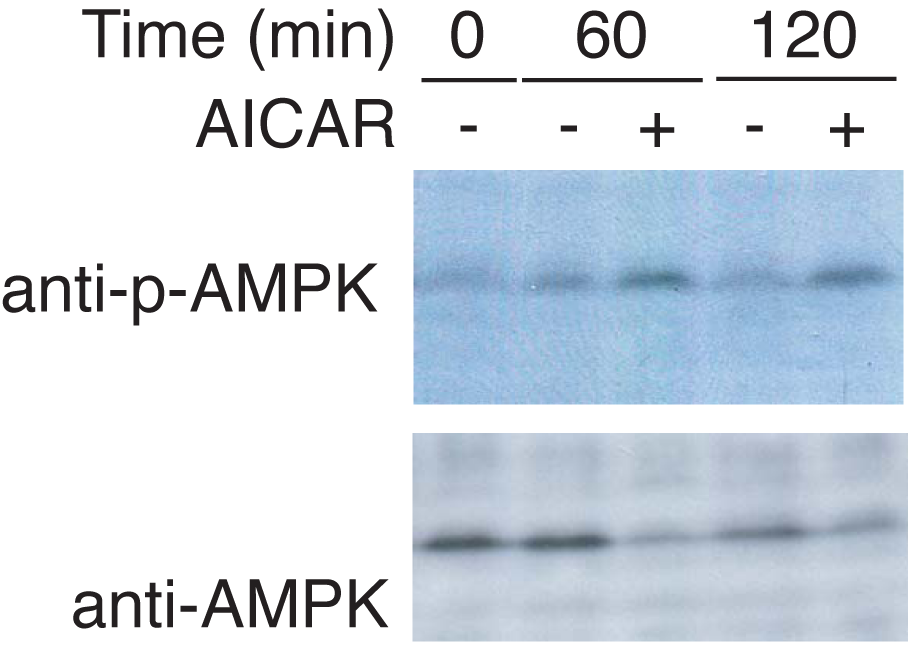

Supplement: Figure S5 — Stimulation of AMPK phosphorylation in the fat body by AICAR. Isolated fat bodies from silkworm were cultured with AICAR (final conc. 0.8 mg/ml) in Grace's insect medium for 0, 60, or 120 min. Fat bodies were homogenized and extracts were prepared. Total AMPK and phosphorylated AMPK were detected by immunoblot analysis. (TIF) [file pone.0018292.s005.tif]

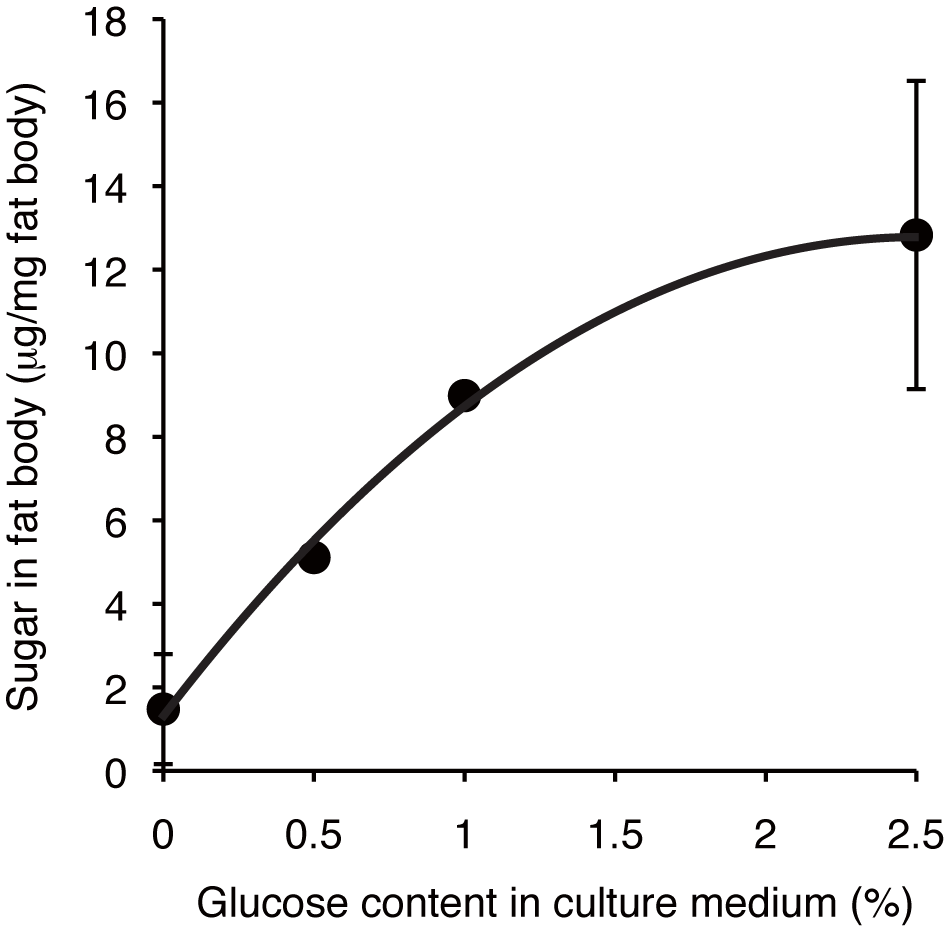

Supplement: Figure S6 — Effect of glucose concentration in the culture medium on total sugar in the fat body. Isolated fat body from silkworms was cultured in Grace's insect medium containing 0%, 0.5%, 1.0%, or 2.5% glucose for 3 h, and the amount of sugar in the fat body was measured. (TIF) [file pone.0018292.s006.tif]

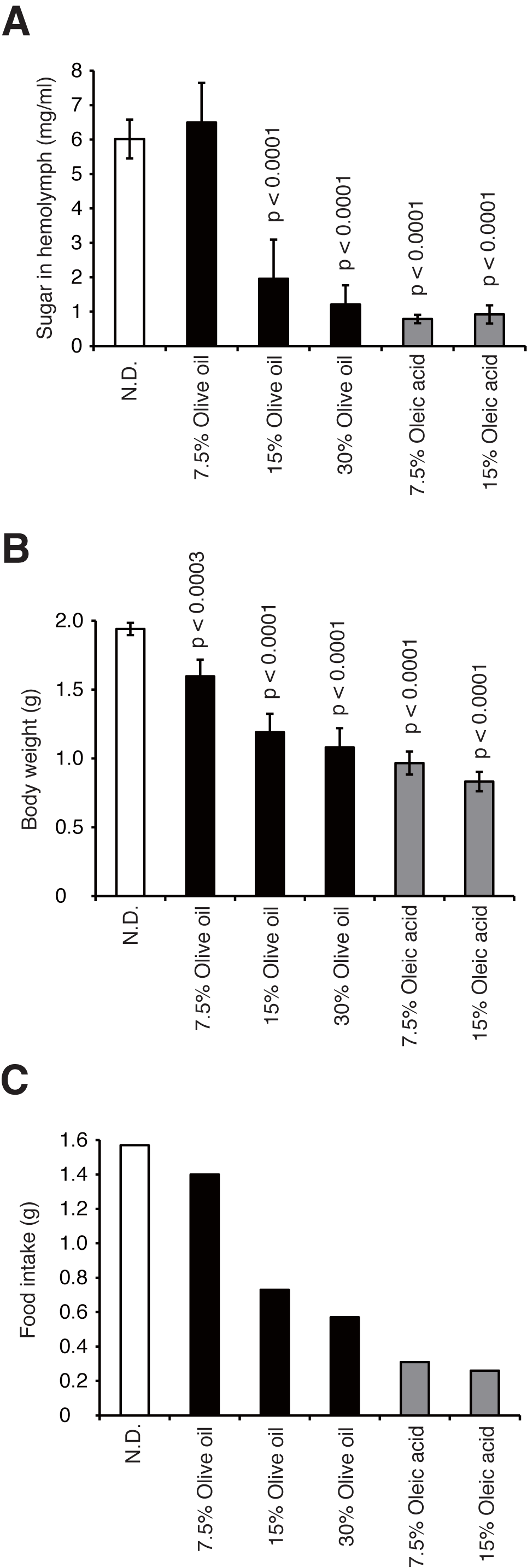

Supplement: Figure S7 — Effect of a high fat diet in silkworms. (A–C) Silkworms were fed a normal diet (N.D.); a 7.5%, 15%, or 30% (w/w) olive oil-containing diet; or a 7.5% or 15% (w/w) oleic acid containing diet for 1 day. Sugar levels in the hemolymph (A), body weight (B), and food intake (C) were determined. n = 5 per group. Data represents mean±SD. The statistical significance of the difference was evaluated using Student's t test. p: P value versus silkworms fed a normal diet (N.D.). (TIF) [file pone.0018292.s007.tif]
